# Augmented contractility of murine femoral arteries in a streptozotocin diabetes model is related to increased phosphorylation of MYPT1

**DOI:** 10.14814/phy2.13975

**Published:** 2019-02-10

**Authors:** Lubomir T. Lubomirov, Hristo Gagov, Mechthild M. Schroeter, Rudolf J. Wiesner, Andras Franko

**Affiliations:** ^1^ Institute of Vegetative Physiology University of Cologne Köln Germany; ^2^ German Center for Diabetes Research (DZD e.V.) Neuherberg Germany; ^3^ Division of Endocrinology Department of Internal Medicine IV Diabetology, Angiology, Nephrology and Clinical Chemistry University of Tübingen Tübingen Germany; ^4^ Faculty of Biology Sofia University St. Kliment Ohridski Sofia Bulgaria; ^5^ Cologne Excellence Cluster on Cellular Stress Responses in Aging‐Associated Diseases (CECAD) Köln Germany

**Keywords:** MYPT1 phosphorylation, streptozotocin‐induced diabetes, vascular tone

## Abstract

Diabetes mellitus (DM) is a metabolic disorder with high prevalence, and a major risk factor for macro‐ and microvascular abnormalities. This study was undertaken to explore the mechanisms of hypercontractility of murine femoral arteries (FA) obtained from mice with streptozotocin (STZ)‐induced diabetes and its relation to the phosphorylation profile of the myosin phosphatase target subunit 1, MYPT1. The immunoreactivity of MYPT1 toward phospho‐MYPT1‐T696, MYPT1‐T853, or MYPT1‐S695, used as a read out for MYPT1 phosphorylation, has been studied by Western Blotting. Contractile activity of FA from control and STZ mice has been studied by wire myography. At basal conditions (no treatment), the immunoreactivity of MYPT1‐T696/T853 was ~2‐fold higher in the STZ arteries compared with controls. No changes in MYPT1‐T696/853 phosphorylation were observed after stimulation with the Thromboxan‐A_2_ analog, U46619. Neither basal nor U46619‐stimulated phosphorylation of MYPT1 at S695 was affected by STZ treatment. Mechanical distensibility and basal tone of FA obtained from STZ animals were similar to controls. Maximal force after treatment of FA with the contractile agonists phenylephrine (10 *μ*mol/L) or U46619 (1 *μ*mol/L) was augmented in the arteries of STZ mice by ~2‐ and ~1.5‐fold, respectively. In summary, our study suggests that development of a hypercontractile phenotype in murine FA in STZ diabetes is at least partially related to an increase in phosphorylation of MLCP at MYPT1‐T696/853. Interestingly, the phosphorylation at S695 site was not altered in STZ‐induced diabetes, supporting the view that S695 may serve as a sensor for mechanical activity which is not directly involved in tone regulation.

## Introduction

Diabetes mellitus (DM) affects millions of people worldwide. This metabolic disorder has two main forms according to the traditional classification: Type 1 DM, which is usually diagnosed during childhood and adolescence and is related to primary damage of *β*‐cells of the pancreatic islet, leading to a reduction in endogenous insulin production; and type 2 diabetes that is characterized by an onset at adulthood and caused by insulin resistance and obesity (Gvazava et al. 2018). DM often coexists with other cardiovascular risk factors and is reported to lead to a poor outcome of many cardiovascular diseases with high social impact, as coronary heart disease, heart and renal failure, ischemic stroke, etc. (Luitse et al. 2012). DM is also often associated with abnormalities in brain microcirculation manifested by an early onset of cognitive decline and development of dementia (Jiwa et al. 2010).

The progression of DM is associated with severe vasculopathies as a typical consequence of hyperglycemia, insulin resistance, and a rise in free lipid concentration (Heinonen et al. 2015). Regarding the risk profile, it has been postulated that type 2 diabetes leads to microvascular complications, whereas type 1 diabetes is a risk factor for macro‐ as well as microvascular complications (Heinonen et al. 2015). In addition, it has been shown earlier that abnormalities in vascular tone regulation induced in diabetes are often manifested as vascular hypercontractility. Thus, augmented reactivity of the large vasculature has been found for various vasoconstrictive transmitter molecules, that is, *α*‐adrenomimetics, 5‐hydroxytryptamine (5‐HT, serotonin), thromboxane A_2_, and PGF_2*α*_ (Kimura et al. 1994; Xie et al. 2006; Matsuo et al. 2011; Matsumoto et al. 2014; Emilova et al. 2016). These studies reported the development of a hypercontractile vascular phenotype in mesenteric, femoral, and renal arteries and in arteria gracilis in different models of type 1 and 2 diabetes in rodents as well as in saphenous veins from diabetes mellitus patients. It has been also reported that the diabetes state impairs intracellular signaling events on the level of the vascular endothelium, leading to endothelial dysfunction manifested by a reduction in vasodilatory response to acetylcholine (Molnar et al. 2005; Elms et al. 2013; Yin et al. 2013). At least part of this impairment and hypercontractile response were related to a reduction in eNOS dimer formation (Molnar et al. 2005). In addition, it has been postulated that the underlying mechanism for this dysfunction is the accumulation of reactive oxygen species (ROS) in vascular endothelium, due to an overexpression of endothelial adhering molecules leading to enhanced monocyte infiltration (Tsao et al. 1998). Furthermore, ROS accumulation has been shown to lead to an augmented release of thromboxane A_2_ after acetylcholine treatment, pointing to the possible role of this contractile autacoid molecule for causing the hypercontractile phenotype of vascular tissue (Taguchi et al. 2014). In line with these findings, increased levels of thromboxane A_2_ and an increase in the expression of thromboxane A_2_ receptor have been reported in murine intrarenal arteries of DM type 2 mice (Kuang et al. 2017). Regarding the complexity of vasculopathies in diabetic conditions, type 1 diabetes is also associated with severe axonopathies and axonal dystrophy, which may also influence vascular tone via vascular nerves (Schmidt et al. 2004). In addition, in a recent study Xie and coauthors suggested that besides endothelial dysfunction and impaired neuronal function, type 2 diabetes might augment contractile responsiveness of aortic cells via direct Ca^2+^ sensitization of smooth muscle. This pathway involves the activation of RhoA/Rho kinase (ROK) and phosphorylation of the C‐kinase‐activated protein phosphatase‐1 (PP1) inhibitor of 17 kDa (PPP1R14A; CPI‐17) and leads to the inhibition of *M*yosin *L*ight *C*hain *P*hosphatase (MLCP) (Xie et al. 2006). These data suggest that diabetes affects all components regulating vascular tone, that is, endothelium, perivascular nerves, and smooth muscle cells and thereby contributes to the development of a complex abnormal vascular phenotype manifested by hypercontractility.

This study has been undertaken to reveal the mechanisms leading to augmented contractility of murine femoral arteries obtained from STZ‐induced diabetic mice, which are characterized by rapid onset of severe hyperglycemia. Using rodent models of STZ induction, during the last two decades a wide spectrum of interaction of diabetes with other diseases was investigated (Franko et al. 2014, 2016), including many studies of the cardiovascular systems (Rosengren 2019). In many experimental models of vascular pathology including DM, the regulatory phosphorylation of MLCP on its targeting subunit MYPT1 has been shown to be the underlying mechanism for hypercontractility (El‐Yazbi et al. 2015; Lubomirov et al. 2017). Therefore, we focused our investigation on basal and agonist‐induced immunoreactivity of MYPT1 in vascular preparations of femoral arteries (FA) obtained from STZ mice and their controls. As MYPT1 phosphorylation has been shown to be an important component of insulin signaling, which in vasculature is responsible for insulin receptor‐mediated vasorelaxation (reviewed in Kiss et al. (2019)), we expected a vascular impairment manifested by hypercontractility in the STZ model of DM. The rationale to use femoral arteries in our study was that in DM patients the impairment in flow has been shown to be a typical disease complication and a leading cause for a nontraumatic surgical amputation of low extremities (reviewed in Shin et al. (2017)).

## Materials and Methods

### Ethical approval

Animal studies were approved by the local government authorities (Bezirksregierung Köln/Landesamt für Natur, Umwelt und Verbraucherschutz, North Rhine Westphalia; **Az.: 8.87‐50.10.37.09.298**) and carried out in accordance with the guidelines of the European Commission (Directive 2010/63/EU) and of the German animal welfare act (TierSchG). The authors declare that represented material is conforming with the principles and standards for reporting animal experiments in “*The Journal of Physiology*” and “*Experimental Physiology*” (Grundy 2015).

### Animals

Streptozotocin treatment was done as described (Franko et al. 2014, 2016). In brief, male, 3 months old C57BL6/N mice (Charles River, Germany) were treated with 60 mg/kg streptozotocin (STZ) for 5 consecutive days or left untreated (controls). Blood glucose was measured in tail blood samples using a glucometer (Bayer, Germany).

### Dissection and stimulation of mouse femoral arteries for measurement of MYPT1 phosphorylation

STZ mice and respective nontreated controls were sacrificed by cervical dislocation 9–16 weeks after STZ injection. The legs were cut off immediately and placed in ice‐cold physiologic salt solution (PSS) with low Ca^2+^, containing in mmol/l: 118 NaCl, 5 KCl, 1.2 NaH_2_PO_4_, 1.2 MgCl_2_, 0.16 CaCl_2_, 10 glucose, and 24 HEPES, pH 7.4 at 37°C. The femoral artery (FA) was isolated distal from its branching point of the arteria gracilis, cut into pieces with nearly equal length (~4 mm length), and mounted on a single wire (40 *μ*m). Thereafter, the vessels were equilibrated in PSS, at 37°C for 30 min and stimulated either by 0.3 *μ*mol/L U46619 or H_2_O (time matched controls; no treatment) for 5 min and directly shock frozen in an acetone/dry‐ice slurry and fixed in acetone/trichloroacetic acid (15%) at −80°C overnight. After fixation, preparations were rinsed twice with acetone on dry ice, left to dry for 5 min, and then homogenized in 30 *μ*L 2xSDS (Laemmli) buffer (in mmol/L): Tris‐HCl 100, dithiothreitol 20, leupeptin 0.002, phenylmethanesulfonyl fluoride (PMSF) 1, aprotinin 40 *μ*g/mL, and sodium dodecylsulfate (SDS) 2%. The lysates were collected, transferred to 1.5 mL reaction tubes, and mixed with equal volumes (30 *μ*L) of 8 mol/L urea (dissolved in H_2_O) and the proteins were extracted for 1 h on ice. Then, the samples were centrifuged at 20,000*g* at 4°C for 10 min, and equal volumes (15 *μ*L) of the resulting supernatants were subjected to SDS‐PAGE (6% or 6–10% gradient). The proteins were transferred to nitrocellulose membrane in a tank blot device and probed with phospho‐specific MYPT1 antibodies directed against either the T696 (dilution 1:30,000; rabbit polyclonal; Millipore, # ABS45), the T853 (dilution 1:10,000; rabbit polyclonal; Millipore, # 36‐003), or the S695 site (dilution 1:5,000; rabbit polyclonal antibody; gift by Dr. Timothy Haystead, Duke University, Durham) or antibodies directed against MYPT1‐total rabbit polyclonal (dilution 1:10,000; # 07‐672, Millipore) or mouse monoclonal (dilution 1:10,000; BD Transduction Laboratories, # 612165). The specificity of commercial antibodies directed against MYPT1‐T696 or MYPT1‐T853 used in our study was characterized earlier (Grassie et al. 2012). The antibody directed against pMYPT1‐S695 was validated by ELISA. pMYPT1‐S695 antibody detected with high‐specificity phosphorylation of the 695 phosphopeptide and those from double phosphorylated or the 696 phosphorylated peptides to a lower degree (data not shown). The immunoreactive signals were visualized by incubation with horse radish peroxidase coupled donkey anti‐rabbit secondary antibody (dilution 1:10,000; # 711‐035‐152, Jackson ImmunoResearch Laboratories Inc., (DIANOVA)) or goat anti‐mouse IG DyLight 680 (# SA5‐10044; Thermo Fisher Scientific) or goat anti‐rabbit IG DyLight 800 (# SA5‐10170; Thermo Fisher Scientific) antibodies (dilution 1:10 000) for 1 h. Equal loading was verified by staining the upper part of the protein gel with Coomassie R‐250 or incubating the nitrocellulose membranes with anti‐MYPT1 total (catalog numbers and dilution as described above). Immunoreactive protein bands were detected with enhanced chemiluminescence (SuperSignal West Dura, Pierce) or Odyssey infrared imaging system (LI‐COR, Lincoln, NE). The optical densities were quantified by densitometric analysis of the chemiluminograms using Phoretix software (Biostep) or Odyssey imaging software (LI‐COR). Phosphorylation was expressed as the ratio of the corrected signals from the respective phospho form of MYPT1 and Coomassie stained Filamin on protein gel or MYPT1 total as in Neppl et al. (2009) and Lubomirov et al. (2018).

### Dissection and mounting of mouse femoral arteries for force measurements

FAs with equal length (~2.0 mm) were isolated as described above and mounted on two wires (40 *μ*m) in a wire myograph (DMT, Denmark, Model 610M). After mounting, the vessels were rinsed four times with PSS containing 1.6 mmol/L Ca^2+^ and bubbled with O_2_. Afterward the temperature was increased/raised to 37°C. Ten minutes after temperature stabilization the vessels were stretched stepwise to their optimal (IC_90_) length determined as in Mulvany and Halpern (1977)and Lubomirov et al. (2017, 2018). Initial basal tone of murine FA was obtained after radial stretch to ~13.3 kPa in PSS, followed by a release with 10% to IC_90_ (90% of internal diameter at which vessels reach wall tension of 13.3 kPa), which was kept over the whole experiment. Basal tone was measured in absolute values in mN 1 and 20 min after adjustment of IC_90_ prior application of contractile agonists. The wall pressure was calculated by the Myograph software using a modified Laplace law in which the pressure was determined using the equation: P = WT/IC/2*π*; where P is pressure, WT is wall tension, determined by measurement of force divided by 2*length of the vascular segment (determined after mounting using a calibrated eyepiece), and IC is internal circumference calculated from the distance between the steel wires plus the known diameter of the mounting wires multiplied by 2+ *π*[IC = (2 + *π*)*40 *μ*m] as in Lubomirov et al. (2017). After equilibration for 20 min, the vessels were incubated with cumulative Phenylephrine (0.1–10 *μ*mol/L) or U46619 (0.01–1 *μ*mol/L) for 5 min each. Reaching the highest concentration the drugs were washed out with PSS. After tone regeneration preparations were incubated with 100 *μ*mol/L N^*ω*^‐Nitro‐L‐arginine (L‐NAME), an inhibitor of nNOS and eNOS, in PSS for 20 min, and a second concentration–response curve was obtained in continuous presence of the NOS inhibitor. Experiments were carried out at 37°C, under continuous bubbling with O_2_ and pH = 7.4.

### Chemicals

The thromboxane analog U46619 was purchased from Tocris; N^*ω*^‐Nitro‐L‐arginine (L‐NAME), phenylephrine, and Streptozotocin from Sigma.

### Statistical analysis

Data are represented as mean ± SD. If not indicated otherwise, *n* is the number of individual experiments, which also equals the animal number. pEC_50_ values were obtained from the individual concentration–response relationships. Statistical comparisons were performed by unpaired *t*‐test or two‐way ANOVA, followed by Sidak's test for multiple comparisons; *P *≤ 0.05 was taken to indicate statistical significance.

## Results

### Glucose values in FA from controls and STZ mice

After injection of STZ, fed blood glucose values were raised by ~2‐fold at 2 weeks compared to those obtained from untreated controls (277.4 ± 31.81 mg/dl in STZ animals compared to 141.6 ± 17.34 mg/dL in controls; *n* = 9). At that time point animals had not lost weight which occurs only at the second month after application (Franko et al. 2014). Data in the animal status are found in Franko et al. (2014).

### Basal phosphorylation of MYPT1 at T696/853 is increased in FA from STZ mice

**Figure 1:**
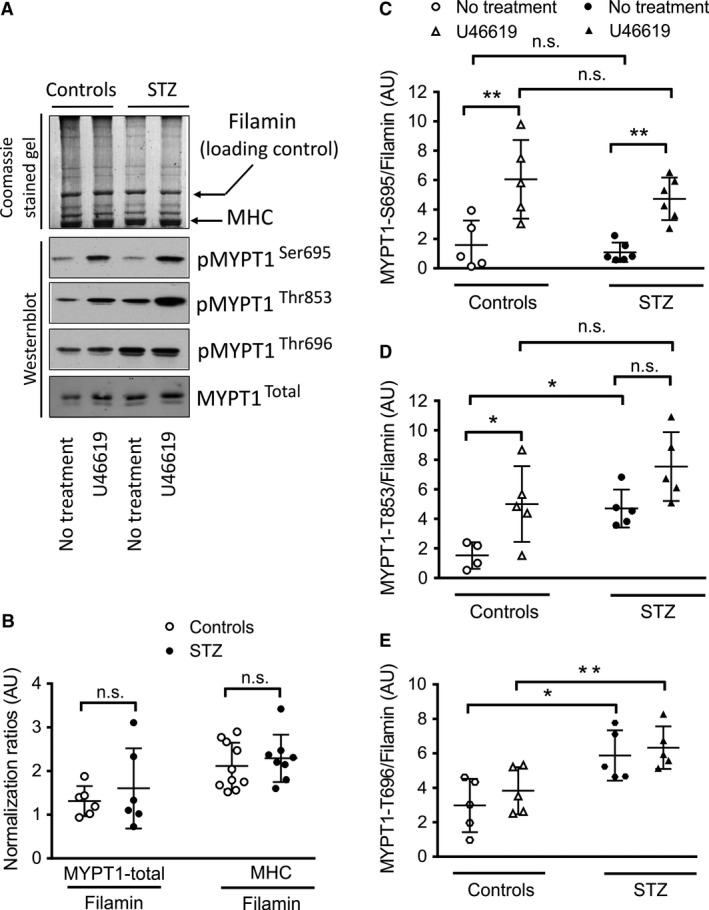
STZ treatment increases MYPT1 phophosphorylation at T696 and T853 in FA, but not at S695. A: Original Western blots probed with phospho‐specific antibodies against MYPT1 phosphorylated at S695 (pMYPT1‐S695), T853 (pMYPT1‐T853), T696 (pMYPT1‐T696), and MYPT1 total in FA of control or STZ‐treated mice. Figure (B) represents the ratio of Coomassie blue R‐250‐stained Filamin on SDS‐PAGE used for normalization of the immunoreactive signal (loading control) toward myosin heavy chain, denoted on the figure as MHC or MYPT1 total. C, D, E: Ratios of pMYPT1‐S695, pMYPT1‐T853, and pMYPT1‐T696 toward Filamin. Phosphorylation was measured either in time‐matched controls (PSS, denoted as “No treatment”) or after 5 min treatment with 0.3 *μ*mol/l U46619 as ** *P*<0.01; * *P*< 0.05; n.s.: not significant; two‐way ANOVA followed by Sidak's multiple comparison post‐test; scatter plot diagrams represent individual values of the ratio of phospho‐protein toward housekeeper ± SD;* n* = 5–6. Results: pMYPT1‐S695: n.s. in PSS (controls) versus PSS (STZ) and in U46619 (controls) versus U46619 (STZ). ** *P*<0.01 in PSS (controls) versus U46619 (controls) and PSS (STZ) versus U46619 (STZ). pMYPT1‐T853: * *P*< 0.05 in PSS (controls) versus PSS (STZ) and n.s. in U46619 (controls) versus U46619 (STZ). * *P*< 0.05 in PSS (controls) versus U46619 (controls) and n.s. in PSS (STZ) versus U46619 (STZ). pMYPT1‐T696: *P < 0.05 in PSS (controls) versus PSS (STZ) and ** *P *< 0.01 in U46619 (controls) versus U46619 (STZ).

We first studied phosphorylation at basal conditions and after U46619 stimulation in FA obtained from STZ and control mice. Immunoreactivity of phospho‐specific antibodies against MYPT1‐T696 or T853 was twofold higher in preparations of FA from STZ‐treated mice than in FA from controls (Fig 1), suggesting that basal phosphorylation is increased in these arteries. As previously shown (Neppl et al. 2009), 5 min after stimulation with the Thromboxan‐A_2_ analog, U46619, 0.3 *μ*mol/L U46619, the immunoreactivity of MYPT1‐T853 increased in both groups but did not reach statistical significance in arteries from STZ mice (Fig. 1). Interestingly, U46619 treatment had no effect on MYPT1‐T696 immunoreactivity in both groups, suggesting that in FA the degree of phosphorylation of this site is independent of the activation of this signaling pathway.

### Phosphorylation of MYPT1 at S695 in FA from control and STZ mice

We also tested the hypothesis whether an increase in phosphorylation of the MYPT1‐T696 site would reflect basal or agonist‐induced phosphorylation of the adjacent phospho‐serine site of MYPT1, S695. Increased S695 immunoreactivity has been shown to go along with the contractile response of U46619 in murine FA and rat brain vasculature (Neppl et al. 2009; Lubomirov et al. 2018). This effect was attributed to NO release and an increase in cGMP concentration. It was even postulated that S695 phosphorylation might serve as an endogenous brake against hyperconstriction (Neppl et al. 2009). However, neither basal nor U46619‐induced increase in MYPT1‐S695 immunoreactivity was altered in FA from STZ mice compared to control animals (Fig. 1).

### Basal tone and contractility toward Phenylephrine or U46619 in FA from control and STZ‐treated mice

We further studied whether the length–tension relationships after radial stretch, the basal tone, and the reactivity of FA toward two contractile agonists, Phenylephrine and U46619, are altered in mice with STZ‐induced diabetes. The force developed after radial stretches did not differ between controls and STZ FA, suggesting that STZ‐induced diabetes did not alter mechanical distensibility of the vessels. We also determined initial basal tone at internal circumference 90 (IC_90_) at 1 or 20 min after the normalization procedure. In control preparations, the tone at 1 min was 4.4 ± 0.2 mN and at 20 min 4.5 ± 0.2 mN, which did not differ from 4.6 ± 0.3 mN and 4.9 ± 0.3 mN, respectively, obtained in FA from STZ mice (*P *> 0.05; *n* = 8–6 preparations from 4 to 3 animals; unpaired test).

**Figure 2:**
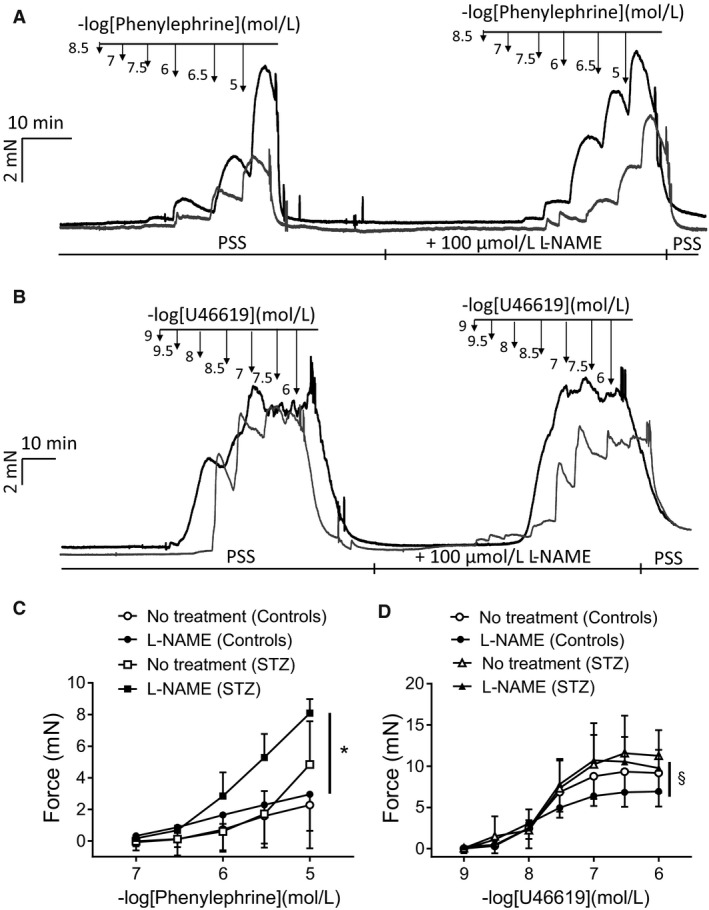
Modulation of Phenylephrine and U46619‐induced contraction in FA of control and STZ‐induced diabetic mice. Original force traces showing the contractile response of control (gray force tracing) and STZ (black) murine FA to increasing concentrations of (A) Phenylephrine (concentration range 0.1–10 *μ*mol/l) or (B) U46619 (concentration range 0.001–3 *μ*mol/l) in the absence or presence of 100 *μ*mol/L L‐NAME. (C, D) Statistical evaluation of the dose–response curves for the effect of Phenylephrine and U46619 on vascular force development. Data represent absolute force in mN ± SD (*n* = 3–4 vessels from 3 to 4 animals; * *P < 0.05 or §*P* = 0.19: two‐way ANOVA, followed by Sidak's test for multiple comparisons).

We further tested the reactivity toward contractile agonists in FA from controls or mice with STZ diabetes. In FA pEC_50_ of U46619 was not altered in STZ‐diabetic model, while the maximal force (*F*_max_) of both agonists tended to increase in FA from STZ mice. Treatment with 100 *μ*mol/L L‐NAME had no effect on basal tone in both groups. However, *F*_max_ in FA from STZ mice compared to controls was increased upon treatment with 10 *μ*mol/L Phenylephrine by ~2.5‐fold (Fig. 2). *F*_max_ of U46619 (1 *μ*mol/L) in FA from STZ mice in the presence of L‐NAME was also ~1.5‐fold greater, but this trend did not reach statistical significance (Fig. 2). L‐NAME also reduced the Hill coefficients in the concentration–response curves of U46619 in FA from controls, but not those from STZ (nH = −1.8 ± 0.4 in absence of L‐NAME vs. −0.9 ± 0.3 in presence of L‐NAME in controls, *n* = 4; −1.6 ± 0.5 vs. −2.0 ± 0.6 in STZ, *n* = 3). As the contractile response to Phenylephrine and U46619 in the systemic circulation greatly depends on the expression pattern of the G*α*_q_ or G*α*_12‐13_ subunits of G proteins in the vascular wall (Wirth et al. 2008), this could explain the observed difference in force generation after STZ treatment. Contractile parameters of femoral arteries of control and STZ mice are presented in Table 1.

**Table 1:** Contractile parameters of FA obtained from control and STZ‐treated mice, stimulated with Phenylephrine or U46619

Group	−log EC_50_ (no treatment)	*F*_max_ (mN)	−log EC_50_ (L‐NAME)	*F*_max_ (L‐NAME; mN)	*n*
Phenylephrine (Controls)	‐	2.3 ± 2.7	‐	3.0 ± 2.2	4
Phenylephrine (STZ)	‐	4.8 ± 2.7^n.s.^	‐	8.1 ± 0.9*	3
U46619 (Controls)	7.8 ± 0.2	8.8 ± 2.8	7.9 ± 0.3	6.9 ± 1.8	4
U46619(STZ)	7.7 ± 0.2	11.3 ± 3.1^n.s.^	7.8 ± 0.1	9.9 ± 2.0^§^	3

Phenylephrine‐induced contraction was not tested at maximal compound concentration, thus the values pEC_50_ were not determined.

n.s.: No significant difference in *F*_max_ in Phenylephrine (Controls) versus Phenylephrine (STZ) in the absence and *P<0.05 for *F*_max_ in the presence of 100 *μ*mol/L L‐NAME (two‐way ANOVA, followed by Sidak's test for multiple comparisons).

n.s.: No significant difference in *F*_max_ in U46619 (Controls) versus U46619 (STZ) in the absence and ^§^ *P* = 0.19 for *F*_max_ in the presence of 100 *μ*mol/l L‐NAME (two‐way ANOVA, followed by Sidak's test for multiple comparisons).

n.s.: No significant difference in pEC_50_ of the preparations from control animals versus STZ has been measured.

## Discussion

Abnormalities in the phosphorylation grade of the myosin filaments are often found to be related to vascular disorders like essential hypertension, diabetes, dementia, etc. (Brozovich et al. 2016). Considerable lines of evidences revealed that not only phosphorylation but also alterations in myosin dephosphorylation might be a primary event for development of disease phenotypes in vascular smooth muscle tissue (Brozovich et al. 2016). Smooth muscle myosin dephosphorylation is catalyzed by MLCP (Dippold and Fisher 2014), which is a trimeric holoenzyme consisting of a 37 kDa catalytic subunit that shows activity toward different intracellular substrates, a 110–117 regulatory targeting subunit (MYPT1), and a 20 kDa subunit with uncertain functions (Grassie et al. 2011). Dephosphorylation of the regulatory light chain of type II myosin by MLCP inhibits interaction of myosin with actin filaments and cross‐bridge cycling and thereby dampens generation and maintenance of active tone (Somlyo and Somlyo 2003). There is a consensus in the literature that the substrate specificity of the MLCP holoenzyme is determined by its targeting subunit MYPT1 either by a direct interaction of the C‐terminal region of MYPT1 with myosin or myosin regulatory light chains, MLC_20_, or via phosphorylation of the central region of MYPT1 by several protein kinases (Hirano 2007; Matsumura and Hartshorne 2008; Grassie et al. 2011). MYPT1 can be phosphorylated at multiple sites: The most important phosphorylation sites that determine the catalytic activity of the enzyme are T696, T853, and S668. The former can be phosphorylated by Integrin‐linked kinase (ILK), zipper‐interacting protein kinase (ZIPK), and Rho‐associated protein kinase (ROK) (Matsumura and Hartshorne 2008), while T853 can be exclusively phosphorylated by ROK (Eto and Kitazawa 2017), and the endmost S668 is exclusively phosphorylated by Protein‐kinase G (PKG) (Yuen et al. 2014). Notably, MYPT1 can be also phosphorylated at sites S695 and S852 by Protein‐kinases A and G (PKA/PKG), whereas other phospho‐serine sites, like S473, have been reported to be phosphorylated during mitosis with a supposed role in cell proliferation (Matsumura and Hartshorne 2008).

Among the phospho‐sites of MYPT1, T696 and T853 have been most extensively studied in the context of the so‐called “phosphorylation paradigm of MYPT1 threonine residues” (Eto and Kitazawa 2017). It is evident that phosphorylation of these residues by ROK causes a reduction in enzyme activity of MLCP (Khasnis et al. 2014). Obviously, the phosphorylation level of MYPT1 at T853 follows most closely fluctuations in force dynamics (Eto and Kitazawa 2017). Studies on isolated vascular preparations reported that an increase in T853 phosphorylation finds its reflection in agonist stimulation (Neppl et al. 2009; Ito et al. 2015; Engholm et al. 2016; Grann et al. 2016), mechanical wall stress in arterioles (Moreno‐Dominguez et al. 2013; Mufti et al. 2015), and NO release inhibition (Neppl et al. 2009; Lubomirov et al. 2018). All these data support the view that phosphorylation at T853 might serve as important mechanosensor involved in normotensive and pathological response of the vasculature. Indeed, abnormal elevation of T853 phosphorylation was observed in diverse models of aging, diabetes, or hypertension (Araos et al. 2016; Watanabe et al. 2016; Abd‐Elrahman et al. 2017; Kuang et al. 2017). In this line, increased ROK‐I expression and a rise in basal MYPT‐T853 phosphorylation were reported in a model of STZ‐induced diabetes (Yin et al. 2013). Supporting all aforementioned observations, our study also reports an elevation of basal phosphorylation of T853 in STZ diabetes.

We also report a nearly twofold increase in phosphorylation of the other phospho‐threonine site of MYPT1, T696 in FA from STZ animals, compared to nondiabetic controls. T696, a site also actively phosphorylated by ROK, is described to be always highly phosphorylated, even in isolated peptides, and less affected by changes in the contractile state of smooth muscle (Eto and Kitazawa 2017). In rat cerebral arteries and bovine trachea, at high [K^+^] or upon stimulation with phorbol‐esters, a slight elevation of T696 has been observed (El‐Yazbi et al. 2015; Gao et al. 2017). Other studies on isolated mesenteric artery smooth muscle cells in culture or isolated basilar arteries from young and old mice reveal the importance of T696 phosphorylation in mediating the vascular response to ROS (Rios et al. 2016; Lubomirov et al. 2017) and in the pathology of contractile responses of cerebral arteries from hypertensive and prediabetic rats (Abd‐Elrahman et al. 2017) or rat retina vessels in diabetes (Rothschild et al. 2017). Notably, the phosphorylation of MYPT1‐T696 affects directly the catalytic activity of the MLCP (Feng et al. 1999), while phosphorylation at MYPT1‐T853 has been shown to be less effective toward MLCP enzyme activity in in vitro studies (Khromov et al. 2009). Furthermore, in vitro replacement of MYPT1 at position T696 by a nonphosphorylatable alanine abrogated the ability of ROK to phosphorylate MYPT1 at T696 (Khasnis et al. 2014), but not when replaced at position T853, questioning the impact of this phosphorylation site on tone regulation. All these findings have been recently confirmed in studies with a transgenic mouse line in which T853 is mutated into nonphosphorylatable alanine, which has negligible impact on contractile function of smooth muscle preparations from urinary bladder, ileum, or trachea (Chen et al. 2015; Gao et al. 2016, 2017). In contrast, the homozygous mutation of T696 into alanine has been shown to diminish ROK‐mediated relaxation of fetal urinary bladder (Chen et al. 2015). In line with these observations we also have shown recently that in heterozygous MYPT1‐T696/A+ mice this mutation reduces the basal tone in young brain vessels (Lubomirov et al. 2018), and impedes development of a hypercontractile phenotype of aged murine basilar artery (Lubomirov et al. 2017). On the other hand, the study of Chen et al. (2015) indicates that tone in the T853A/+ mutant can be lowered with a ROK inhibitor but not in the T696A/+ mutant. From these results, the authors suggest that the ROK inhibitor lowers tone not via dephosphorylation of T853 but rather by affecting actin filament availability which is also ROK dependent. ROK has been shown to be involved in increased actin polymerization and contribution to tone generation in pressurized brain arteries (Moreno‐Dominguez et al. 2013). Thus, at present, the relevance of T853 phosphorylation for the hypercontractility of diabetic vessels is still elusive and represents an interesting route for future studies.

This work also supports the view that STZ diabetes does not alter serine phosphorylation of MYPT1 at S695, independently of augmented phosphorylation of the threonine T696 phospho‐site of MYPT1. This phospho‐site, primarily described to serve as an endogenous sensor of PKA and PKG, does not change the catalytic activity of MLCP, but rather prevents the inhibitory phosphorylation of the adjacent threonine phospho‐site, T696 (Wooldridge et al. 2004). These findings prompted several groups to hypothesize that serine and threonine phosphorylations of MYPT1 are mutually exclusive and thus prephosphorylation at serine might interrupt the phosphorylation of the adjacent threonine site (Wooldridge et al. 2004; Nakamura et al. 2007). In support to this hypothesis, a rise in S695 phosphorylation in brain and femoral arteries has been shown after agonist stimulation with U46619 (Neppl et al. 2009; Lubomirov et al. 2018). However, a later study of Somlyo`s group reported that the alanine mutation of S695 did not prevent 8‐Br‐cGMP‐induced relaxation (Khromov et al. 2012). However, this did not invalidate the hypothesis for a sensor function of MYPT1‐S695, but rather pointed out that MYPT1‐S695 may not participate directly in the regulation of the contractile response. Because MYPT1 has been found to localize in the cleavage furrows of two dividing daughter cells, together with MLCK and ROK, it was also hypothesized that alteration of MLCP activity might be of great importance in the nucleus for acto‐myosin interaction accompanying cell cleavage (Matsumura and Hartshorne 2008). In summary, our study suggests that development of a hypercontractile phenotype in murine FA in STZ diabetes is at least partially related to a nearly twofold increase in basal, inhibitory phosphorylation of MLCP at MYPT1‐T696/853. Interestingly, our study shows that the phosphorylation of serine site S695 was not changed in STZ diabetes, supporting the view that these phosphorylation events may serve as a sensor for mechanical activity and are not directly involved in tone regulation.

### Study limitations

Although this study represents a correlation between phosphorylation of MLCP targeting subunit MYPT1 and hypercontractile phenotype of FA at STZ‐induced diabetes, some limitations must be acknowledged. Although STZ‐induced diabetes resembles to a great extent type 1 diabetes, no requirement for insulin therapy is an important point for cautious interpretation of the results from animal studies and their translation to humans. Moreover, our study is limited as we investigated the effect of STZ‐induced diabetes on vascular tissue only in a certain time window after the onset of hyperglycemia (9–16 weeks after STZ treatment). Other studies on rodent models show differences in contractile response comparing long and short time points after onset of diabetes (Chang and Stevens 1992; Molnar et al. 2005; Abd‐Elrahman et al. 2017). In addition, as our study focused on the importance of MYPT1 phosphorylation, in order to achieve optimal separation of MYPT1, we selected a SDS‐PAGE system with an acrylamide concentration (see “Methods”) in which the MLC_20_ and CPI‐17 polypeptide chains were lost (Lubomirov et al. 2006), thus not allowing the investigation of the phosphorylation of these proteins.

### Perspectives

Given that T696 phosphorylation is twofold increased in vascular tissue in STZ diabetes and that the mutation of T696 into alanine (T696A/+ mutants) is able to diminish the development of a hypercontractile phenotype of aged murine basilar arteries (Lubomirov et al. 2017), this work could encourage future studies on diabetes in T696A/+ mutants. Additional support for conducting such studies is the elevated phosphorylation of T696 in the saphenous vein of diabetic patients (Matsuo et al. 2011).

## Conflict of Interest

The authors declare no potential conflict of interest.

## Supporting information




**Figure S1**. MYPT1 phophosphorylation at S695, T853, and T696 in FA from STZ and controls normalized to MYPT1 total
